# Development and validation of a Bayesian survival model for inclusion body myositis

**DOI:** 10.1186/s12976-019-0114-4

**Published:** 2019-11-07

**Authors:** Gorana Capkun, Jens Schmidt, Shubhro Ghosh, Harsh Sharma, Thomas Obadia, Ana de Vera, Valery Risson, Billy Amzal

**Affiliations:** 1 Novartis Pharma AG, Postfach, CH-4002 Basel, Switzerland; 2 Department of Neurology, University Medical Center Göttingen, Göttingen, Germany; 3 Novartis, Hyderabad, India; 4 LA-SER Europe, Paris, France; 5 Certara, Paris, France

**Keywords:** Inclusion body myositis, Chronic disease, Rare disease, Prognosis, Bayesian model, Survival modelling, Bridging

## Abstract

**Background:**

Associations between disease characteristics and payer-relevant outcomes can be difficult to establish for rare and progressive chronic diseases with sparse available data. We developed an exploratory bridging model to predict premature mortality from disease characteristics, and using inclusion body myositis (IBM) as a representative case study.

**Methods:**

Candidate variables that may be potentially associated with premature mortality were identified by disease experts and from the IBM literature. Interdependency between candidate variables in IBM patients were assessed using existing patient-level data. A Bayesian survival model for the IBM population was developed with identified variables as predictors for premature mortality in the model. For model selection and external validation, model predictions were compared to published mortality data in IBM patient cohorts. After validation, the final model was used to simulate the increased risk of premature death in IBM patients. Baseline survival was based on age- and gender-specific survival curves for the general population in Western countries as reported by the World Health Organisation.

**Results:**

Presence of dysphagia, aspiration pneumonia, falls, being wheelchair-bound and 6-min walking distance (6MWD in meters) were identified as candidate variables to be used as predictors for premature mortality based on inputs received from disease experts and literature. There was limited correlation between these functional performance measures, which were therefore treated as independent variables in the model. Based on the Bayesian survival model, among all candidate variables, presence of dysphagia and decrease in 6MWD [m] were associated with poorer survival with contributing hazard ratios (HR) 1.61 (95% credible interval [CrI]: 0.84–3.50) and 2.48 (95% CrI: 1.27–5.00) respectively. Excess mortality simulated in an IBM cohort vs. an age- and gender matched general-population cohort was 4.03 (95% prediction interval 1.37–10.61).

**Conclusions:**

For IBM patients, results suggest an increased risk of premature death compared with the general population of the same age and gender. In the absence of hard data, bridging modelling generated survival predictions by combining relevant information. The methodological principle would be applicable to the analysis of associations between disease characteristics and payer-relevant outcomes in progressive chronic and rare diseases. Studies with lifetime follow-up would be needed to confirm the modelling results.

## Background

For chronic, slowly progressive, rare and orphan diseases there is often only limited understanding of the full disease burden and natural disease evolution. These are important elements in informing clinical research, designing clinical studies and educating healthcare providers on unmet patient needs, as well as for assessing the value of new treatment options.

Outcomes management aims to “help patients, payers, and providers make rational medical care-related choices based on better insight into the effect of these choices on the patient’s life” [1]. A reliable characterisation of disease burden and evolution often needs a large population of patients and long-time longitudinal follow-up, which may be unavailable. As a consequence, the link between disease-related characteristics and outcomes is not well established for a number of uncommon and slowly progressing illnesses.

In the absence of long-term data for a specific disease, novel applications of statistical modelling may be used to synthesise and integrate existing heterogeneous data. The present communication describes a modelling study to assess the predictive potential of disease-related characteristics and outcomes, using inclusion body myositis (IBM) as a case of a rare, slowly progressive, chronic disease.

IBM is a rare and debilitating progressive chronic muscle disease, primarily affecting those aged over 50 years [2, 3]. There are sparse available data on the long-term natural history of IBM or on predictors of poor outcome for subjects with disease. As a consequence, no consensus exists on whether disease characteristics are associated with increased mortality. Only a handful of studies have included mortality analyses and the findings are not consistent [4–6].

We developed a predictive modelling and simulation tool we refer to as a “bridging model”, that bridged from early disease characteristics to mortality in IBM patients. The bridging model relied on data from the literature as well as from existing observational studies across a range of chronic diseases and elderly patients. A Bayesian framework was used to synthesise and integrate the data. For external validation, the survival hazard in the predictive model was compared with survival in IBM cohorts reported in two published studies [4, 5]. A sensitivity analysis was performed to identify variables with the greatest predictive value and a hypothesis of influence of IBM on mortality was suggested. This approach could be applied to other diseases where information is lacking linking disease and important outcomes.

## Methods

### Overall approach

**Fig. 1:**
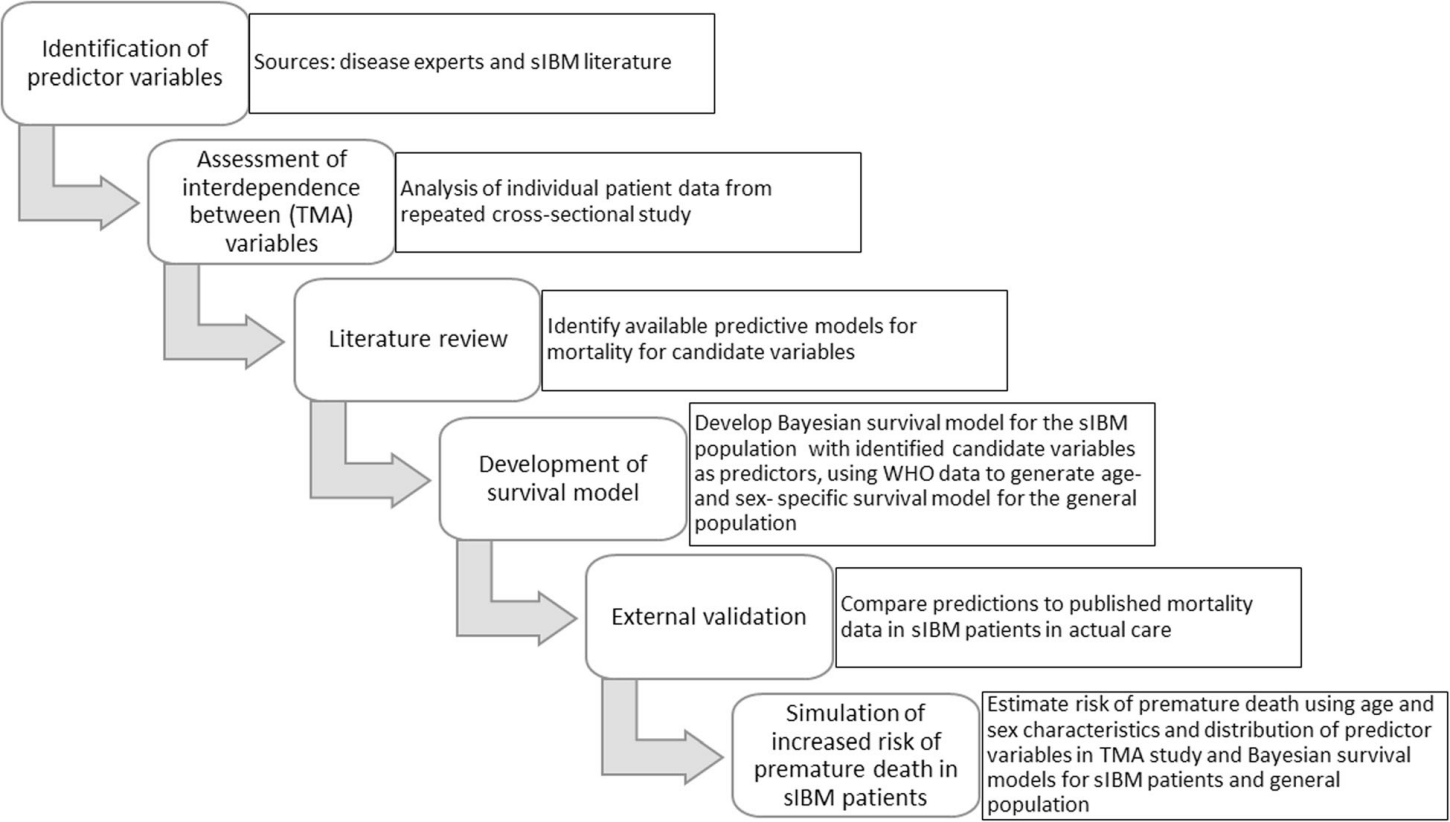
Schematic representation of the steps taken to develop the model presented in the current work. The individual steps are described in the text

The modelling study followed a step-wise approach outlined in Fig. 1.

Firstly, candidate IBM characteristics that may be associated with mortality were identified by disease experts and from the IBM literature. Possible interdependence between these variables in IBM patients were assessed using existing cross-sectional, non-interventional, observational patient-level data from the US collected during The Myositis Association (TMA) annual conferences in 2013 and 2014 (data available on file). The informed consent allowed for between-years patient matching. Only summary statistics needed for modelling was provided to the investigators. As patients may have been included multiple times over the lifetime of the registry, only the earliest record available for each patient was used (“baseline” patient data). In the following, we refer to this study as the TMA dataset.

For the selected variables (see Additional file 1 for a list), an extensive literature review was conducted to identify available predictive models describing an association between selected variables and mortality in neurodegenerative diseases with elderly populations. For each variable, hazards ratios (HRs) for the association with survival were extracted along with the prevalence (categorical variables) or distribution (continuous variables) of each predictor in both control and IBM populations. If associations were analysed in ≥1 publication for a selected variable, a Bayesian method for meta-analysis was applied to quantitatively synthesise and analyse the findings as described below.

From the results, a Bayesian survival model for the IBM population was developed using the selected clinical variables as predictors as detailed below. For external validation, the predictions of the selected models were compared with published mortality data in IBM patients in actual care [4, 5] and the most parsimonious model was chosen.

After validation, the model was used to estimate the increased risk of premature death in IBM patients using the distribution of outcomes in the TMA study. As a control, age- and gender-specific survival curves for the general population in Western countries were generated based on World Health Organisation (WHO) data.

Details of these steps are given below.

### Identification of candidate variables of premature mortality in IBM patients

Candidate predictors of premature mortality in IBM patients were identified from a recent Delphi survey publication by 13 IBM experts [6] as well as in discussions with members of the Delphi group and other clinical experts.

### Assessment of interdependence of variables

Patient-level data were obtained by trained researchers who administered questionnaires that included demographic items, clinical outcomes (number of falls in the past month, 6-min walking distance (6MWD [m]), presence of dysphagia, ambulatory status), other clinical characteristics (age at first symptoms, age at first diagnosis, use of walking aids or devices) and patient questionnaires including the sporadic inclusion body myositis physical functioning assessment (sIFA) [7, 8]. Data from 102 unique consenting patients were obtained using a secure, server-based data collection application or paper forms. The data set was used to assess the possible interdependence of the identified variables.

No mortality data were recorded. Dysphagia was captured with the sIFA scale items 8 and 9 (“swallowing liquids” and “swallowing solids” VAS scale). Presence of dysphagia was derived from the “swallowing” score that had the highest dispersion. “Swallowing solids” was recorded as a binomial variable to reflect the presence (score ≥ 1) and absence (score = 0) of dysphagia. The extracted patient data were tested for collinearity using the Spearman rank correlation coefficient.

No individual-level data were available for presence of aspiration pneumonia, which is a predictor of mortality in IBM [6]. This functional outcome was imputed at the patient level based on published materials [9] with a baseline prevalence of 1% and an increased risk-ratio of ~ 9.1 (95% CI: 7.5–11.2) when a patient from the TMA dataset suffered from dysphagia.

### Literature review of survival predictors

An extensive literature review was conducted to identify analyses of mortality increases associated with changes in candidate variables. The search strategy is provided in Additional file 1. In order to maximise the relevance of results to IBM patients, the search was restricted to studies reporting on populations with neurodegenerative disease, or general/elderly populations to serve as a reference. A structured review protocol (detailed in Additional file 1) was developed to identify and extract the relevant publications. Study quality and risk of bias was assessed on prospective vs. retrospective design, sample size, measurement errors and missing data.

The following data were extracted from the publications: patient numbers, age distribution, gender distribution, mortality, mortality rate, mean or median duration of follow-up, HR for mortality linked to a specific outcome, and the proportion of patients with aspiration pneumonia among patients with dysphagia. From the TMA dataset, information was included on patient numbers, mean and standard deviation for 6MWD [m], and the proportion of patients with presence of falls, dysphagia or in wheelchair.

Effect-size estimates were extracted from all identified papers (in the form of the natural logarithm of the HR) together with the variance of the estimate, derived from the HR and 95%-confidence interval (95% CI), assuming a normal distribution of effect sizes. Denoting *β*_*i*_ = log(*HR*_*i*_) the effect size in study *i* and *L*_*i*_ the lower bound of the associated 95% CI, the variance was computed from the formula:

$$ Var\left({\beta}_i\right)={\left(\frac{\upbeta_{\mathrm{i}}-\log \left({L}_i\right)}{1.96}\right)}^2 $$

A random effect model was used to account for between-study heterogeneity. Specifically, denoting *δ*_*i*_ the random effect associated with study *i* assumed to come from a normal distribution with mean *d*, the underlying effect size and variance *τ*^*2*^ (between-study variance) would be:

$$ {\displaystyle \begin{array}{c}{\beta}_i\sim Normal\left({\delta}_i, Var\left({\beta}_i\right)\right)\\ {}{\delta}_i\sim Normal\left(d,{\tau}^2\right)\end{array}} $$

The prior for the pooled effect-size *d* was normally distributed with mean 0 and variance 10^5^ while the prior for the between-study variance was uniform [0; 10].

Three Markov chains of 50,000 iterations were performed, with the first 25,000 iterations as burn-in (R, OpenBUGS, R2OpenBUGS) and convergence assessed on the last 25,000 iterations, by visual inspection of the Markov chains with the R-hat Gelman-Rubin statistic as a numerical indicator.

A separate literature review was conducted to identify publications on mortality in IBM patients.

### Development of Bayesian survival model for the IBM and general populations

Based on the literature review, the reference hazard function in the general population was modified to account for the effect of dysphagia, falls and 6MWD [m]. Exploration of the TMA dataset did not show any clinically and statistically significant correlation between the functional outcomes other than correlation between use of wheelchair and walking distance. When generating virtual cohorts, the 6MWD [m] variable was always set to 0 for patients who used a wheelchair for the 6MWD [m] test.

With *i* denoting the index of a given individual, the saturated model used the following parameterisation for *λ*:

$$ {\lambda}_i=\exp \left({\beta}_{\mathit{\operatorname{int}},i}+{\beta}_{dys}\ast dy{s}_i+{\beta}_{AP}\ast A{P}_i+{\beta}_{falls}\ast {falls}_i+{\beta}_{6 MW D}\ast \Delta 6 MW{D}_i+{\beta}_{wc\mathrm{h}}\ast wc{\mathrm{h}}_i\right) $$

where *β*_*predictor*_ is the coefficient associated with the corresponding predictor variable, and *predictor*_*j*_ is the value of this predictor variable in individual *i*. More details are provided in Additional file 2 and codes (R software) are available in Additional file 3.

In regard to the reference survival model, this new parameterisation implicitly assumed that the general population was comparable with the control populations from studies used to extract hazard data.

Sex- and age-adjusted survival curves from the WHO for Western countries served as reference survival curves in the general population. All-cause mortality rates and counts were extracted from the WHO Life expectancy public database repository for European countries [10]. These data present death rates in 5-year incremental age groups and by sex.

Death rates were fitted to an interval-censored Weibull survival model for each sex, providing two continuous functions that fitted the step-functions extracted from the WHO data (further details on the model are provided in Additional file 2). To improve the goodness of fit, we restricted the curve to individuals > 45 years old, reflecting the usual age range of IBM patients. A mortality null model (containing only age and sex covariates, with mortality risk based on WHO data) was simulated, to assess differences in mortality rate between the general and IBM populations.

### Model selection and external validation

The best model was selected and validated by comparing the predicted mortality rates with those of IBM patient populations for which mortality data have been published. Potential publications enabling such external validation were identified from the literature review.

Virtual cohorts of IBM patients were generated from the survival model matching the age and sex distributions in the two publications. The distribution of the predictor variables were derived from the TMA dataset.

For the virtual cohorts, age and sex were taken from the populations in the validation studies. Height was included from the sex-specific distribution in the TMA dataset. Weight was predicted using a linear regression model with age, gender and height as predictors. The “dysphagia” and “falls” predictor variables were calculated from binomial distributions with probabilities equal to those observed in the TMA dataset. For “lower body severity” status, a multinomial distribution with probabilities equal to that observed in the TMA dataset was used, with the assumption that all individuals with “severe” lower body impairment were wheelchair-bound. “6MWD” [m] was predicted using a linear regression model using age, height and weight as predictors. “Aspiration pneumonia” was generated from a binomial distribution using baseline probabilities reported for the general population and adjusted by the (sex- and age-adjusted) relative risk for dysphagia patients reported by Altman et al [9].

Virtual patients were assumed to have survived at least until their age at “baseline” estimated from the age distributions reported in the validation publications. The sex ratio was determined from the observed proportion of male patients in the validation publications. Binary variables (falls, dysphagia, aspiration pneumonia and wheelchair status) were estimated from binomial distributions *B*(*n*, *p* = *p*_*i*_) where *p*_*i*_ is the observed proportion of individuals experiencing outcome *i* in the TMA dataset. The continuous variable (6MWD [m]) was derived from the linear regression model for the 6MWD in IBM patients, i.e. significantly influenced by age.

Model selection was performed on the basis of model parsimony and coverage statistics, i.e., whether credible intervals (CrI) for the percent of patients dying in a simulated cohort included the value observed in the reference study [5] and the difference between the median predicted survival rate and that reported in validations studies. All possible univariable and multivariable models were considered, with covariates selected from 6MWD [m], dysphagia, falls, wheelchair status and aspirational pneumonia. The models were then ranked by their coverage statistics. Subsequently, a stepwise forward process was used to expand to models with three and four variables. The final model was then selected as the most parsimonious achieving the best coverage and mean prediction of the reference study mortality data.

### Simulation of increased risk of premature death in IBM patients

Starting from the estimates of *β*_*predictor*_ of the final model, the distribution of the HR for death in a cohort of IBM patients similar to that from the TMA dataset was computed as:

$$ HR={e}^{\beta \ast \Delta X} $$

where *ΔX* is the difference in value between the predictor *X* in IBM patients and in the general population of the same age and sex.

Finally, overall HR_IBM_ was estimated comparing the IBM condition with the general population (with 95% CI) and the posterior probability of an HR_IBM_ > 1 derived from the survival model by simulating a virtual cohort. Accounting for the uncertainty of the coefficient estimates and for the distribution of functional outcomes in the IBM population, a virtual cohort of 10,000 patients was generated. First, age and sex of each patient were derived from the distributions found in the TMA dataset; subsequently the distribution of the two dichotomous outcomes in IBM patients was derived from binomial distributions with probabilities equal to that observed in the TMA dataset. The distribution of 6MWD [m] values in IBM patients was derived from the linear regression model described above.

The general population was assumed not to suffer from falls nor dysphagia (i.e. *X*_*dysphagia*, *general*_ = 0 and *X*_*falls*, *general*_ = 0). Finally, the distribution of corresponding 6MWD [m] in the general population was sampled using data from the available literature [11, 12], resulting in a modulating effect of 6MWD [m] scaled to the extent of its variation between sound individuals and patients suffering from neurodegenerative diseases.

For each patient the value of λ was sampled using the Bayesian framework along with a reference value *λ*∗ corresponding to that of an age- and gender-matched individual with mortality corresponding to the WHO data. The overall HR for death was therefore *HR* = exp {*λ* − *λ*^∗^}.

## Results

### Identification of candidate IBM variables predictive of premature mortality

The following candidate variables were identified: 6MWD [m] [13], presence of falls [14], presence of dysphagia [9, 14–16], presence of aspiration pneumonia and being wheelchair-bound. The only continuous covariate was 6MWD, typically reported in meters and for which hazard was provided for a 1-m decrease.

### Literature review and synthesis

**Fig. 2:**
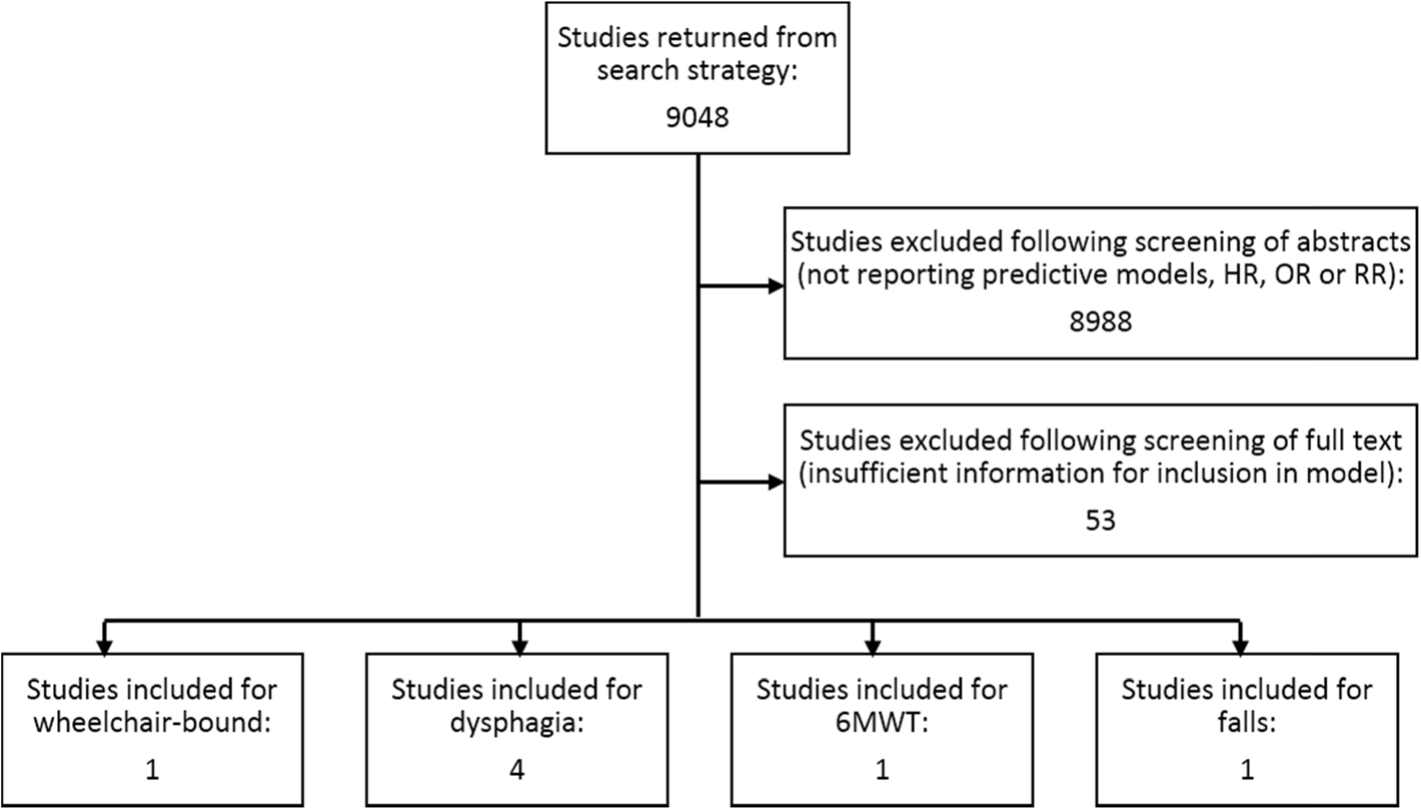
Flow chart for the literature review

**Fig. 3:**
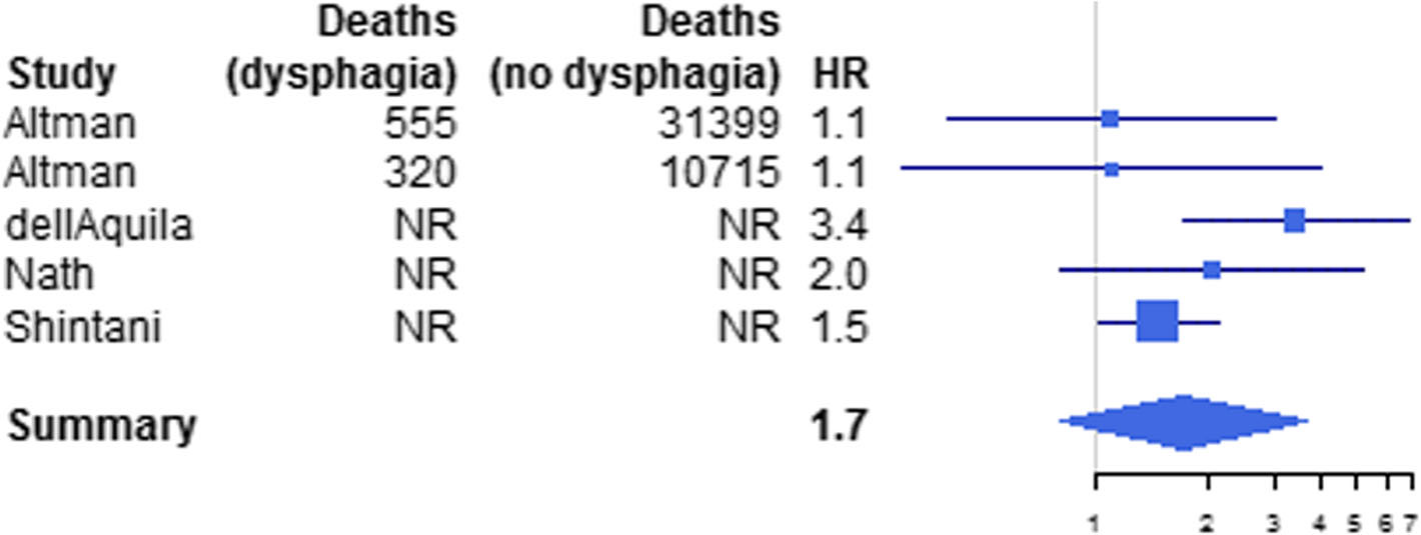
Forest plot of hazard ratios and 95% CI used in the quantitative synthesis and analysis of dysphagia data

Figure 2 illustrates the results of searches and the screening process for the structured review. A total of 5 studies were included following study screening [9, 13–16]. One publication was identified for 6MWD [m], presence of falls, and being wheelchair-bound, supplying a HR for premature death. For dysphagia, 4 publications were identified [9, 14–16]; the effect-size d had a posterior mean of 0.54 and variance 0.38, yielding a pooled HR of 1.72 (95% CrI 0.81; 3.65) (Fig. 3). One additional study was identified regarding aspiration pneumonia [17] based on a separate supplemented search (see Additional file 1) providing RR for premature death. For 6MWD [m], we estimated that IBM patients could walk in median 281 m less compared to the general population (95% CrI: 157–399).

### Assessment of interdependence of variables

Exploring independence between variables in the TMA data set, no obvious trend was found, except for a negative correlation between wheelchair status and both 6MWD [m] and presence of falls. These findings supported the assumption of an overall additive hazard for the considered predictor variables and a need to account for interactions between the three that were correlated.

### Model selection and validation

Two validation studies were identified which reported on mortality in IBM patients. The publication by Cox et al. [5] included 64 patients in The Netherlands with a median 12.5-year follow-up. The study by Benveniste et al. [4] included 136 patients from two centres, with a median follow up of 2.5 years. Both works reported patient age and sex.

A mortality null model (containing only age and sex covariates, with mortality risk based on WHO data) was simulated, to assess differences in mortality rate between general and IBM populations. For a virtual cohort adjusted to the population in the study by Cox et al., the mean mortality rate over 12.5 years in the null model was 57.5% compared with 71.9% in the study. For a virtual cohort adjusted to the population in the Benveniste et al. study, the mean null model mortality over a 2.5 year follow-up period was 19.8% compared with 18.4% in the study cohort (Table 1). As the model adjusted for age, sex and symptoms duration at baseline, we assumed that the disparity in case of this study comes from a large variability in follow-up times, the distribution of which might not necessarily be normal. Median follow-up times were 31 months, with an interquartile range (IQR) 5–75 months. For this reason, only the study by Cox et al. was included in the model selection process.

**Fig. 4:**
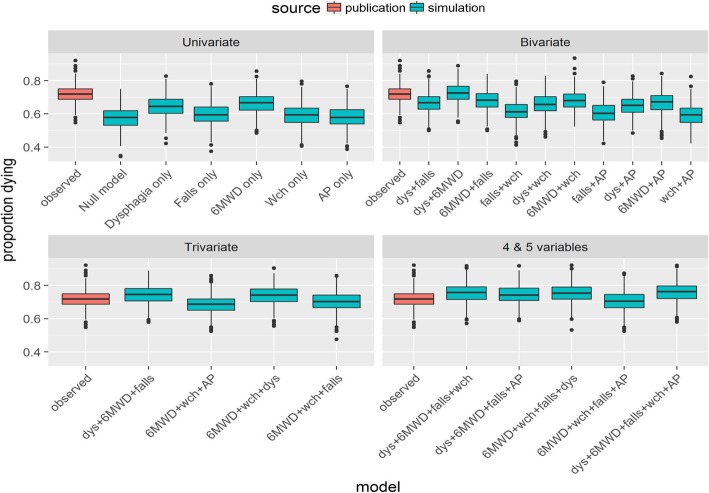
Model selection: Boxplots of proportion of patients who died across models (from top left to bottom right: univariate, bivariate, trivariate and with 4 & 5 variables). Null model: Model without covariates, AP: Aspiration Pneumonia, Wch: Wheelchair bound, Dys: Dysphagia

Among the univariable, bi-, tri- four- and five-variable models simulated with the Cox data characteristics, the most parsimonious model with the greatest coverage and closest alignment with the published data for the probability of death was the one including 6MWD [m] and presence of dysphagia (Fig. 4).

### Estimation of premature mortality in IBM patients

The reference hazard function in the general population was modified to account for the presence of dysphagia and decrease in 6MWD [m]. As the two covariates were independent, the total HR attributable to IBM vs the general population was derived by a simple multiplication of the two. The hazard ratio for death in IBM patients vs. the general population was estimated at 4.03 (95% prediction interval 1.37, 10.61) with a 99.8% posterior probability of being > 1. The model suggests that IBM shortens patients’ lives by approximately 12 years. Presence of dysphagia and reduced 6MWD [m] contributed to impaired survival with the associated HR 1.61 (95% CrI 0.84–3.50) and 2.48 (95% CrI 1.27–5.00) respectively.

**Table 1:** Summary of observed and predicted deaths with cohorts composed as in the validation papers and predictor variables distributed as observed in the TMA dataset

Validation study	N	Study Duration (median)	Observed deaths	Predicted deaths (base-case model)	Predicted deaths (saturated model)
Cox et al. [5] (overall)	64	12	72% [61%; 83%]	61% [57%; 66%]	76% [64%; 85%]
Benveniste et al. [4]	136	2.5	18% [12%; 25%]	14% [11%; 17%]	35% [27%; 43%]

## Discussion

### Results and implications

Using a Bayesian predictive modelling approach based on information from the literature in comparable populations, data from WHO and cross-sectional data from the disease population of interest, we developed an exploratory bridging model of how different disease-related variables may be associated with premature mortality in rare and insufficiently researched chronic diseases. The model used IBM as a disease example but the concept was designed to be applicable more generally to other rare diseases for which important outcomes data are lacking or difficult to obtain. The final model provided realistic predictions validated on European Union IBM patients with mild to severe symptoms and 12.5 years of median life expectancy. Compared with the validation study [5] the mean predicted mortality numbers matched the observed mortality closely, although the coverage statistic was low.

Informed decision-making involves combining the knowledge arising from clinical expertise, patient preferences, and research evidence within the context of available resources. For rare diseases, such information is often sparse, particularly for long-term outcomes. This deprives physicians of important insights as well as reduces the ability of policy makers to make informed policy decisions around unmet needs, access and reimbursement. In the absence of disease-specific data, novel applications of statistical, modelling and simulation tools may help to identify associations between variables and outcomes.

Variable and model selection is a critical step of model development and is determined by the data availability. In the current model, the variables were selected for their relevance to patients with IBM and for known associations with poor outcomes such as survival in elderly populations and/or in other neuro-degenerative diseases [9, 13–16]. Variables ranged from presence of dysphagia, a clearly definable clinical condition, to being wheelchair bound or 6MWD [m]. The latter is a functional limitation, which reflects a major degree of disability and may be seen as an outcome in itself, as it is driven by a number of underlying variables not addressed directly.

### IBM as representative disease

IBM is a suitable disease for the development and validation of the Bayesian predictive model for several reasons. It is a rare and slowly progressive, but serious and potentially life-threatening illness, with few data available to inform therapy decisions, possibly reflecting challenges to obtaining relevant information from traditional data sources [6]. In the absence of evidence, there is no consensus among experts on the impact of the disease on a number of outcomes including mortality. Some complications of the disease, e.g., pneumonia arising from dysphagia, are considered to be associated with greater risk of death compared with healthy individuals [4, 5]. In a recent Delphi study, there was widespread agreement, based on the clinical experience and patient records available to a panel of expert physicians, that IBM patients with bulbar dysfunction, dysphagia and oropharyngeal involvement have a shortened lifespan compared with patients without IBM [6]. This seems especially relevant in elderly patients with IBM [18].

Our model suggested an increased mortality risk from IBM, with a HR of 4.03 for death vs. the general population, attributable to decreased mobility (measured by decrease in 6MWD [m]) and presence of dysphagia. A 12-year reduction in life span with the disease was predicted. The results should be viewed as exploratory and would need to be further validated and refined with long-term data on the disease. They do, however, support the Delphi panel view that IBM may impact mortality, which could influence how the disease is viewed by the clinical community.

A comparison may be made with multiple sclerosis (MS). A few decades ago consensus was that this disease did not impact mortality to a significant extent and that life expectancy was shortened by only a few months [19]. In the last decade, a number of real-world studies have revealed a major impact of MS on mortality, with patients facing a decrease in life expectancy of 5–10 years compared with the general population [18, 20, 21]. The availability of statistical tools similar to our model may help predict such outcomes in other diseases and steer research towards more focused investigations of mortality.

### Strengths and weaknesses of the bridging model

The model makes relatively conservative predictions, as it does not control for the presence in the WHO-derived reference curves of IBM patients and of patients experiencing dysphagia or reduced mobility from other causes. If the presence of other disorders affected the risks in the control population, this would be expected to reduce the effect sizes attributed to IBM-related variables in the model.

In the general population dysphagia is associated with increasing age, with prevalence rates 30–40% in elderly people in long-term care facilities [22]. As we controlled for age, the confounding impact of age-related disorders can be expected to be small. The most common relevant neurological disorders are Parkinson’s Disease and MS with incidence rates around 1 in 350 in the general population [21, 23]. Other neuromuscular disorders tend to be rare, from 1 in 3500 male births (Duchenne’s muscular dystrophy) to 1 in 7500 (myasthenia gravis) or less [23].

As with all predictive models, there are limitations to the design. Data to inform the model were scarce and with gaps. Input data were derived from heterogeneous sources: the TMA dataset used to generate associations between outcomes and distributions of age/sex in IBM populations was based on a US cohort whereas the WHO survival curves used to define mortality baseline risk were based on data from unspecified “Western countries”. For a number of variables included in the model there are limited supporting data, often from studies with small sample sizes. The observational nature of the studies, their secondary [9, 13] or retrospective [15, 16] natures and small sample size [15] in combination with measurement errors and presence of missing data can lead to biased estimates of HR for mortality which would ultimately lead to the biased estimates of sIBM-related mortality risks. However, it should be noted that the purpose of the work was not a precise estimate of mortality in sIBM, but to conduct a feasibility study of how bridging models can fill evidence gaps, raise awareness and motivate further research by generating new hypotheses. We also note that the HR estimates obtained with the model were similar to those reported by the Delphi panel [6].

The strongest supporting body of data was available for dysphagia, for which information from 4 publications could be synthesised and analysed. For 6MWD [m] and falls, only one publication each was available. In addition to the extrapolation from diseases other than IBM, this lack of data and limited quality of underlying studies represents a weakness in the model.

The publication used to validate and finalise the model was a small study that focused on IBM patients in the Netherlands [5]. Additional heterogeneity may have arisen from differences in disease stages and severity between the populations. Information on severity could be used to refine the model. Some of the predictors were also constrained to binary (e.g. dysphagia / no dysphagia) although different levels of severity would have increased the clinical and statistical relevance. The TMA dataset can only inform on the distribution of the main functional outcomes in the IBM patient population to the extent that it is representative; moreover it did not include mortality data. As the data were collected during congresses, it may represent a healthier-than average patient population.

If more information around rare chronic diseases becomes available in the future, it would be possible to update and refine the model. There is an on-going rapid accumulation of real-world data on patients and treatments world-wide, driven by electronic data management and the adoption of internet technologies. Greater use of these tools would enable physicians to gather more data in future on the impact of IBM from registries and similar sources of real-world information as well as patient-level sources. This would provide the opportunity to apply the new model to larger datasets.

## Conclusions

This Bayesian bridging model provided an estimate of association between different disease related variables and premature mortality in IBM. The mortality in IBM was greater than in the general population with an HR of 4.03; the presence of dysphagia and impaired 6MWD [m] contributed with HRs of 1.61 and 2.48, respectively. The validation showed good agreement between model estimations and published data. The approach might be applicable to other rare, progressive chronic diseases to help identify clinical variables associated with poor outcomes, which would aid decisions regarding therapy and guide policy makers. However, larger cohort studies with sufficient follow-up would be needed to confirm hypotheses generated from modelling approaches.

## Supplementary information

**Additional file 1.** Search strategy and review protocol (DOCX 22 kb)

**Additional file 2.** Accelerated failure time model (DOCX 59 kb)

**Additional file 3.** Codes for analysis (R software) (DOCX 32 kb)

## Data Availability

The datasets for the study are available from the corresponding author on reasonable request.
