# Supplementary material for: Development and validation of a Bayesian survival model for inclusion body myositis
Source: Theor Biol Med Model. 2019 Nov 7;16:17. doi: 10.1186/s12976-019-0114-4 (PMC6836518; doi:10.1186/s12976-019-0114-4)
Supplement: Supplementary file 1 — Additional file 1. Search strategy and review protocol (DOCX 22 kb) [file 12976_2019_114_MOESM1_ESM.docx]

**Additional file 1: Search strategy and review protocol**

The following data sources were searched as part of the review:

- EBM Reviews - Cochrane Database of Systematic Reviews 2005 to May 2015

- EBM Reviews - Database of Abstracts of Reviews of Effects 2nd Quarter 2015

- EBM Reviews - Cochrane Central Register of Controlled Trials May 2015

- EBM Reviews - Cochrane Methodology Register 3rd Quarter 2012

- EBM Reviews - Health Technology Assessment 2nd Quarter 2015

- EBM Reviews - NHS Economic Evaluation Database 2nd Quarter 2015

- Embase 1996 to 2015 July 9

- Ovid MEDLINE(R) without Revisions 1996 to June Week 4 2015

- Ovid MEDLINE(R) In-Process & Other Non-Indexed Citations July 9, 2015

All searches were restricted to English language studies conducted in a human

population. The table details the search terms used to identify studies for the review.

**Literature review search strategy**

|  | Query string |
| --- | --- |
| 1 | ([(Mortal* OR fatal*).ab,ti.] OR exp accidental death/ OR exp brain death/ OR exp “cause of death”/ OR exp dying/ OR exp fatality/ OR exp heart death/ OR lethality/  OR exp sudden death/ OR exp suicide/ OR exp “time of death”/ OR exp mortality/ OR survival/ or exp cause specific survival/ or exp disease free survival/ or exp disease specific survival/ or exp event free survival/ or exp failure free survival/ or exp life expectancy/ or exp long term survival/ or exp overall survival/ or exp post treatment survival/ or exp progression free survival/ or exp recurrence free survival/ or exp short term survival/ or exp survival factor/ or exp survival prediction/ or exp survival rate/ or exp survival time/) |

| 2 | ((predict.ab,ti.) OR exp prognosis/ OR exp survival/ OR *nomogram/ OR exp |
| --- | --- |
|  | regression analysis/ OR (correlation.tw.) OR Relation* OR (regression analyses.tw.)) |
| 3 | exp dysphagia/ |
| 4 | exp Fall/ |
| 5 | (exp wheelchair/ OR (ambulatory OR non-ambulatory OR "non ambulatory").tw.) |
| 6 | (6 minute walk distance OR 6-minute walk distance OR 6MWD OR 6 minute walk Test OR 6-minute walk Test OR 6MWT).mp. |
| 7 | 1 AND 2 AND 3 |
| 8 | 1 AND 2 AND 4 |
| 9 | 1 AND 2 AND 5 |
| 10 | 1 AND 2 AND 6 |

Studies were included if they met the following inclusion criteria:

- Reports on one of the following outcomes of interest:

- - - - Dysphagia
      - Falls
      - Being wheelchair bound
      - 6 minute walking test

- Reported on the association between one of the above outcomes of interest and survival (or mortality)

- Study was conducted within one of the following populations:

- - - - Patients with neurodegenerative disease
      - Elderly population
      - General population

The review was subsequently supplemented with key word searches for studies reporting

on aspiration pneumonia to inform that particular outcome in the saturated version of the

model.

Studies were excluded if they were not published in English or if they failed to meet the

inclusion criteria above
