# Supplementary material for: Development and validation of a Bayesian survival model for inclusion body myositis
Source: Theor Biol Med Model. 2019 Nov 7;16:17. doi: 10.1186/s12976-019-0114-4 (PMC6836518; doi:10.1186/s12976-019-0114-4)
Supplement: Supplementary file 2 — Additional file 2. Accelerated failure time model (DOCX 59 kb) [file 12976_2019_114_MOESM2_ESM.docx]

**Appendix 2: Accelerated failure time model**

We used an Accelerated Failure Time of Weibull-type model to build an all-cause mortality survival function as a base-case scenario. The model was developed in a Bayesian framework so that it could yield posterior estimates of variables of interest, which were later incorporated into extensions of the model.

Denoting *T* the survival time, the model can be written as:

$$\log\left( T \right)=\mu+\sum_{i} \alpha_{i}X_{i}+\sigma W=\sum_{i} {\alpha'}_{i}{X'}_{i}+\sigma W$$

where *μ* is a constant (intercept), *α_i_* are a set of regression coefficients, *X_i_* are explanatory covariates, and *W* is the extreme value distribution. For readability, we chose to incorporate *μ* in the vector of regression coefficients now denoted by *α^’^* (thus adding a column of 1 to the matrix of covariates, now denoted by *X’*). Furthermore, by using the notation:

$$\left\{ \begin{aligned} v=\frac{1}{\sigma} \\ \beta=-\frac{\alpha^{'}}{\sigma}=-\alpha v \\ \lambda=e^{-\frac{\alpha^{'}X^{'}}{\sigma}}=e^{\beta X^{'}} \end{aligned} \right.$$

In this model, λ is parameterized in term of explanatory covariates and associated coefficients. The survival time *T* therefore has a Weibull distribution with density:

$$f\left( t \right)=\lambda vt^{v-1}*e^{-\lambda t^{v}}$$

where:

- The hazard function is $h\left( t \right)=\lambda vt^{v-1}$
- The survival function is ${S\left( t \right)=e}^{-\lambda t^{v}}$

Using this parameterization allows for interpreting *β* coefficients as the natural logarithm of hazard ratios, *i.e.* $exp(\beta_{i})$ is the hazard ratio of death for a 1-unit increase in covariate *X’_i_*. The *α* coefficients introduced earlier can be interpreted as the natural logarithm of acceleration factors, *i.e.* the logarithm of event-time-ratios for a 1-unit increase in corresponding covariates.

One model was fit for males and another for females, yielding no estimate of a gender effect. This approach was chosen because the model incorporating a gender effect failed to properly capture the overall shape of the step-function derived from WHO data. Denoting *i* the gender of the individual, the exact parameterization used was:

$$\left\{ \begin{aligned} f_{i}\left( t \right)=\lambda_{i}v_{i}t^{v_{i}-1}*exp(-\lambda_{i}t^{v_{i}}) \\ \lambda_{i}=exp(\beta_{int,i}) \end{aligned} \right.$$

This model resulted in the estimate of four parameters: the shape and intercept, for both genders, which are summarized within Table 1 and illustrated in Figure 1.

Table 1: Parameter estimates in the base-case survival model.

| Fitted curve | Parameter | Mean | SD |
| --- | --- | --- | --- |
| Male | *v_M_* | 6.824 | 0.2668 |
|  | *β_int, M_* | -29.53 | 1.17 |
| Female | *v_F_* | 7.440 | 0.1956 |
|  | *β_int, F_* | -32.43 | 0.86 |

The fit allowed for smoothing the step-function derived from WHO data, as seen below.

|  |
| --- |

Figure 1: Survival curves for the general western populations by gender, as derived from WHO data and fitted Weibull regression models.
