# Supplementary material for: Development and validation of a Bayesian survival model for inclusion body myositis
Source: Theor Biol Med Model. 2019 Nov 7;16:17. doi: 10.1186/s12976-019-0114-4 (PMC6836518; doi:10.1186/s12976-019-0114-4)
Supplement: Supplementary file 3 — Additional file 3. Codes for analysis (R software) (DOCX 32 kb) [file 12976_2019_114_MOESM3_ESM.docx]

**Appendix 3: Codes for analysis (R software)**

Codes for doing analysis are available below as attached file (written in R software).

Corresponding data are available upon request to the author.
